# Supplementary material for: A new species of Nanhsiungchelys (Testudines: Cryptodira: Nanhsiungchelyidae) from the Upper Cretaceous of Nanxiong Basin, China
Source: PeerJ. 2023 May 30;11:e15439. doi: 10.7717/peerj.15439 (PMC10237181; doi:10.7717/peerj.15439)
Supplement: Supplemental Information 3 [file peerj-11-15439-s003.docx]

A new species of *Nanhsiungchelys* (Testudines: Cryptodira: Nanhsiungchelyidae) from the Upper Cretaceous of Nanxiong Basin, China

Yuzheng Ke^1^, Imran A. Rahman^2, 3^, Hanchen Song^1^, Jinfeng Hu^1^, Kecheng Niu^4, 5^, Fasheng Lou^6^, Hongwei Li^7^, Fenglu Han^1^

^1^ School of Earth Science, China University of Geosciences (Wuhan), Wuhan, Hubei, China.

^2^ The Natural History Museum, London, UK.

^3^ Oxford University Museum of Natural History, Oxford, UK.

^4^ State Key Laboratory of Cellular Stress Biology, School of Life Sciences, Xiamen University, Xiamen, Fujian, China

^5^ Yingliang Stone Natural History Museum, Nan'an, Fujian, China

^6^ Jiangxi Geological Survey and Exploration Institute, Nanchang, Jiangxi, China

^7^ Guangdong Geological Survey Institute, Guangzhou, Guangdong, China

Computational fluid dynamics

Computational fluid dynamics (CFD) is an useful tool for simulating flows of fluids and their interaction with solid surfaces (*Rahman, 2017; Gibson et al., 2021*). This basic principle involves transforming the Navier–Stokes equations corresponding to flow problems into algebraic equations and solving them using certain numerical methods at finite discrete moments and spatial nodes (grids) (*Guo et al., 2019*). Recently, CFD techniques have been used in paleontology to quantitatively assess the habits and ecology of a wide range of extinct organisms (*Shiino et al., 2009; Shiino & Kuwazuru, 2010; Shiino et al., 2012; Kogan et al., 2015; Liu et al., 2015; Dynowski et al., 2016; Gutarra et al., 2019; Rahman et al., 2020; Gibson et al., 2021; Song et al., 2021; Gutarra & Rahman, 2022; Gutarra et al., 2022*). In our research, CFD was used to evaluate the drag forces of turtles (*Nanhsiungchelys yangi* and two ‘hypothetical turtles’, see below) in water, and the simulations were performed in the software COMSOL Multiphysics (v. 5.6).

Digital modelling

Considering the close relationship between *Nanhsiungchelys yangi* and *N. wuchingensis* in our phylogenetic hypothesis, we reconstructed a full 3-D model of *N. yangi* (Fig. 1A–C) by referring to the holotype of *N. wuchingensis* (IVPP V3106). The 3-D reconstruction was created using the in-built geometry tools in COMSOL. The main structures of the turtle model were created with simple shapes (e.g. ellipsoids and cylinders). In addition, interpolation curves were drawn in Plane Geometry, which were then further extended into faces. Lastly, several fillets were added to create rounded corners on the 3-D geometries. This model was scaled to an entire carapace length of 1.0 m based on well-preserved specimens of *Nanhsiungchelys wuchingensis* (IVPP V3106) and *N.* sp. (SNHM 1558) which are 0.87 to 1.11 m in entire carapace length (*Hirayama et al., 2009; Tong & Li, 2019*). Considering extant turtles swim with their heads and necks stretching from the shells to breath and look for food (*Schaffer & Schaffer, 2008; Behera et al., 2019*), an idealized head and neck were added to the model to give the model a total length of 1.25 m.

In addition, we constructed two hypothetical 3-D model of turtles without the anterolateral processes on the carapace for comparison, whose total length was also 1.25 m (Fig. 1D–I). Firstly, we cut the anterolateral processes of *Nanhsiungchelys yangi* vertically, thus the new model is ‘hypothetical turtle Ⅰ’. The anterior part of the shell and body of the hypothetical turtle Ⅰ is blunt, parallel to some extant sea turtles (e.g. *Chelonia mydas*) and tortoises (e.g. *Chelonoidis hoodensis*) (*Zhou & Li, 2013; Zhou & Zhou, 2020*). Secondly, we use a more curved surface to cut the anterolateral processes of *Nanhsiungchelys yangi*, and the new model becomes hypothetical turtle Ⅱ. Therefore, the anterior part of the shell of the hypothetical turtle Ⅱ is more streamlined, similar to the condition of most freshwater turtles (e.g. *Mauremys sinensis*) (*Zhou & Li, 2013*).

For each model of length *L*, a cylindrical computational domain was created, whose upstream length was 3×*L*, downstream length was 10×*L*, and radius was 5× the maximum width of the model, following *Gutarra et al. (2019)* (Fig. 1J). As these models are bilaterally symmetrical, only half of the turtle models and cylinders were used in simulations in order to reduce the computation time.


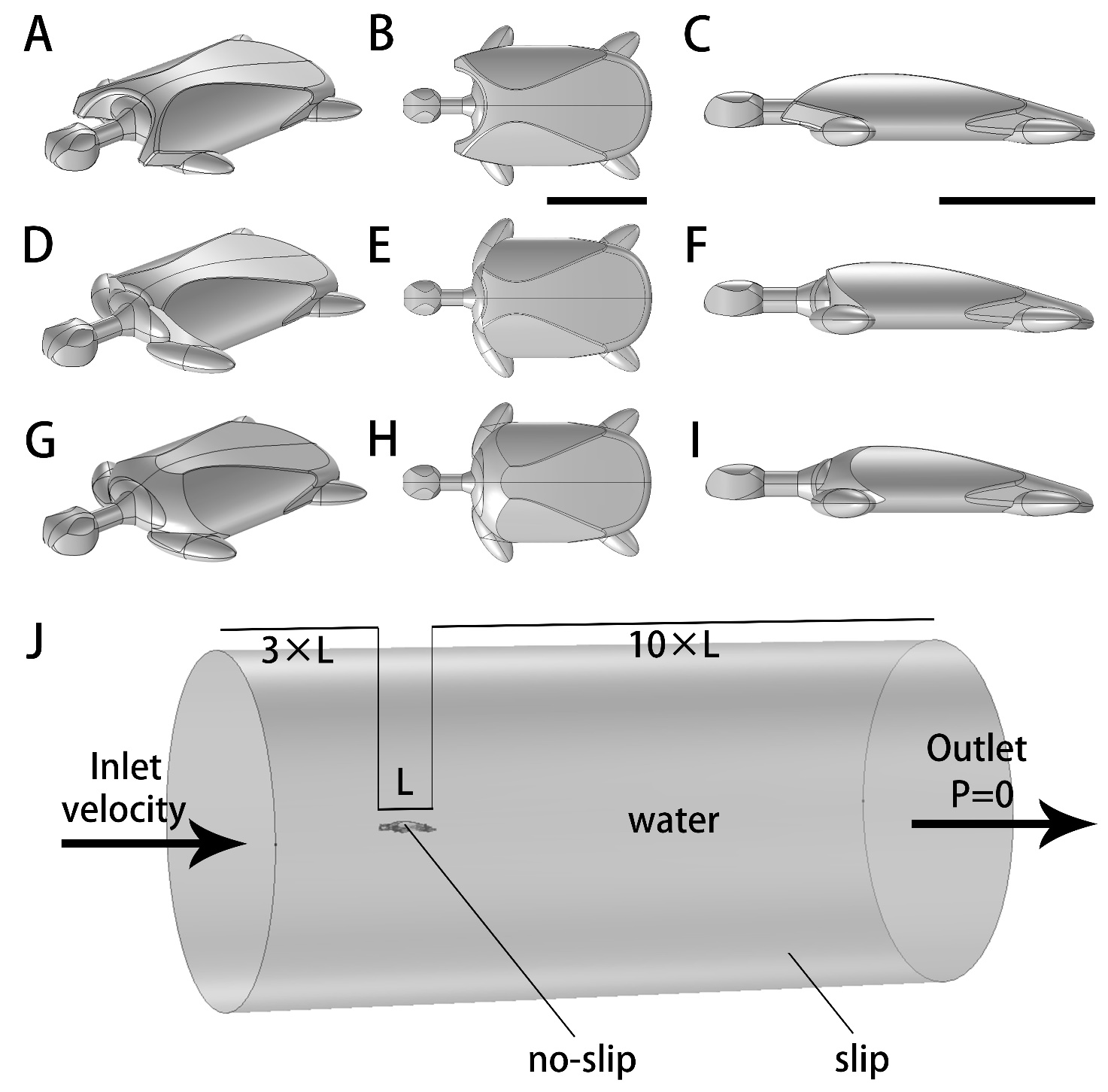


Figure 1. Three-dimensional digital models of *Nanhsiungchelys yangi* (A–C), hypothetical turtle Ⅰ (D–F), hypothetical turtle Ⅱ (G–I), and the computational domain used for computational fluid dynamics simulations (J). Scale bars equal 0.5 m.

Fluid properties and boundary conditions

Parts of the domain inside the cylinder, surrounding the turtle model, were assigned the material properties of water using the built-in materials library in COMSOL. The upstream end of the cylinder was set as the inlet (turbulent intensity is 0.05, with the flow velocity specified here) and the downstream end of the cylinder was set as the outlet (pressure condition is static, pressure specified as 0 Pa, and suppress backflow is selected) (Fig. 1J). The swimming speeds of extinct *Nanhsiungchelys* and their similar-sized living relatives (e.g. *Rafetus swimhoei*) are unknown, therefore the other distant relatives with a large body size are compared here. The modal and maximum swimming speeds of the extant leatherback sea turtle *Dermochelys coriacea* (which has a curved carapace length from 1.45 to 1.69 m) are known to range from 0.56–0.84 m s^-1^ and 1.9–2.8 m s^-1^, respectively (*Eckert, 2002*). It is highly unlikely the swimming speeds of nanhsiungchelyids were faster than leatherback sea turtles due to the lack of paddle-like limbs, and we therefore simulated swimming speeds of 0.6 m s^-1^, 1.0 m s^-1^, 1.4 m s^-1^, 1.8 m s^-1^, 2.2 m s^-1^, and 2.6 m s^-1^ in our study.

The flow regime was characterized using the dimensionless Reynolds numbers (*Re*) (*Reynolds, 1883; Gibson et al., 2021*):

$Re=\frac{\rho UL}{\mu}$ (1)

where *ρ* is the density of water (1000 kg/m^3^), *U* is the velocity of water flow (m s^-1^), *L* is the model’s maximum width (m), and the *μ* is water’s dynamic-viscosity coefficient. The *Re* of our simulations was ~4.75×10^5^ to ~2.06×10^6^, which falls within the range of turbulent flow (i.e. *Re*＞1×10^4^) (*Gutarra et al., (2019)*, supplementary information). As a result, the *k-ε* turbulence model was used in all our simulations, which is robust, economizes on computational cost, and is known to be reasonably accurate for a wide range of turbulent flows (*Adkins & Yan, 2006*). Slip boundary condition was assigned to the side of the cylindrical computational domain, and no-slip boundary condition was assigned to the surface of the 3-D turtle models.

Mesh size and computation

The domains were meshed using free tetrahedral elements, with prismatic boundary layer elements inserted along the interface between the turtle model (Fig. 2). A stationary solver was used to compute the steady state flow patterns, with the segregated iterations terminated when the relative tolerance reached 1×10^-4^.

To evaluate the effect of mesh size on the CFD results, sensitivity tests were conducted with different meshes. Using an inlet velocity of 1.0 m s^-1^, three different mesh sizes (‘normal’ to ‘finer’) were used for each of the models, and this showed that the drag forces and drag coefficients did not change significantly (Table 1, Fig. 3). As a result, the finer mesh was selected and used in our analyses, which was composed of numerous smaller cells and thus could most accurately represent the flow (*Rahman, 2017; Gibson et al., 2021*).

Drag forces were computed for each model based on surface integration. Drag coefficients (*C_D_*) were then calculated using the following equation (*Rahman, 2017; Gibson et al., 2021*):

$C_{D}=\frac{{2F}_{D}}{\rho U^{2}A}$ (2)

where *F_D_* is the drag force (N), *ρ* is the density of water (1000 kg/m^3^), *U* is the velocity of water flow (m s^-1^), and *A* is the characteristic area (m^2^). Please note that the final results of drag forces (Tables 1, 2 and Figs. 3, 4) are twice the calculated results, this is because only half of the turtle models and cylinders were used in simulations. Moreover, streamlines around the turtle models (Fig. 5) were visualized.


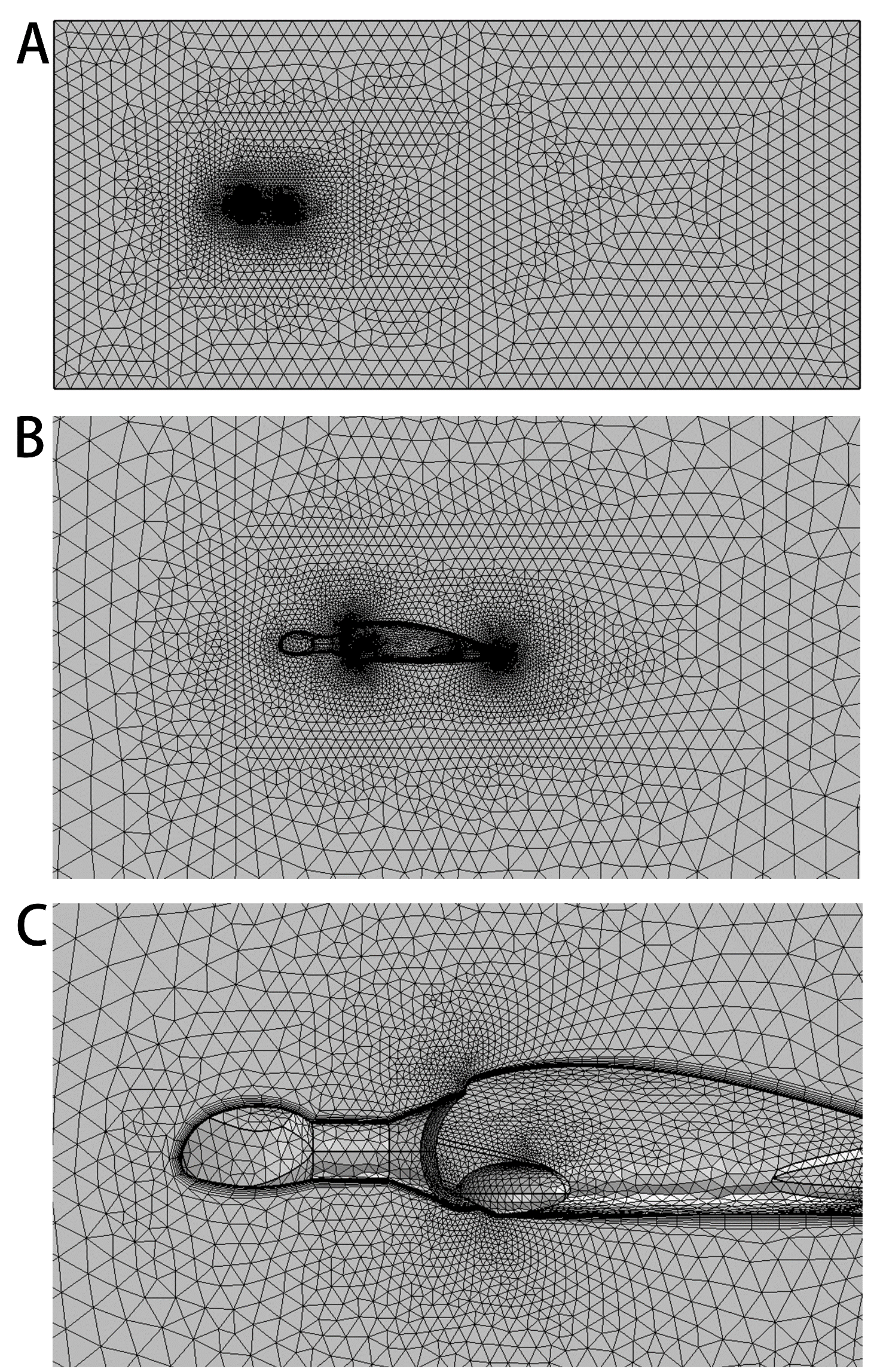


Figure 2. Mesh used in CFD simulations. (A) Mesh used in the whole computational domain. (B) Details of the mesh near the turtle model. (C) Details of the mesh near the anterior of the turtle model.

Table 1:

Drag forces and drag coefficients obtained for different mesh sizes for three-dimensional digital models of *Nanhsiungchelys yangi* and two hypothetical turtles.

|  | *Nanhsiungchelys yangi* | | | Hypothetical turtle Ⅰ | | | Hypothetical turtle Ⅱ | | |
| --- | --- | --- | --- | --- | --- | --- | --- | --- | --- |
| Mesh size | Number of elements | Drag force (N) | Drag coefficient | Number of elements | Drag force (N) | Drag coefficient | Number of elements | Drag force (N) | Drag coefficient |
| Normal | 380495 | 22.312 | 0.372 | 316015 | 28.058 | 0.468 | 377724 | 20.430 | 0.341 |
| Fine | 644382 | 20.892 | 0.348 | 495692 | 27.088 | 0.451 | 613812 | 19.356 | 0.323 |
| Finer | 1431064 | 19.176 | 0.320 | 1006082 | 25.826 | 0.430 | 1256156 | 17.974 | 0.300 |


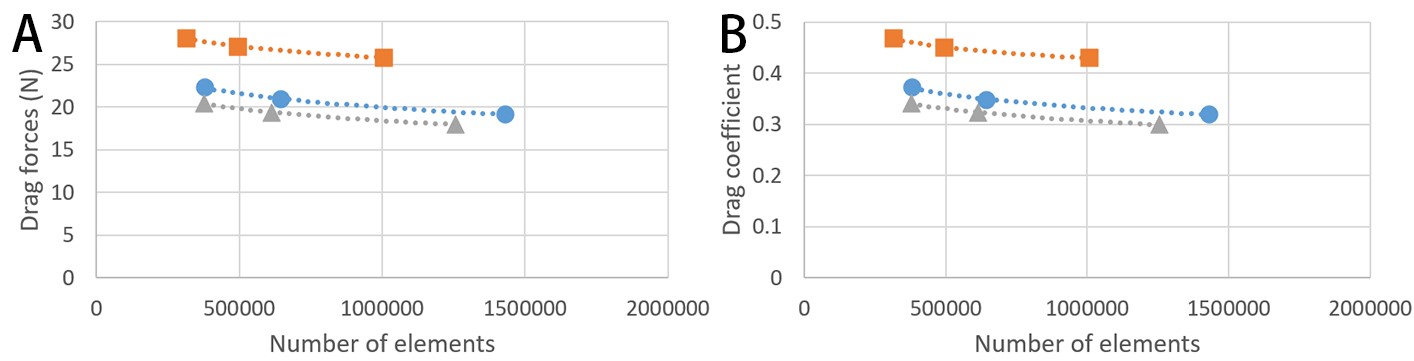


Figure 3. Comparison of drag forces (A) and drag coefficients (B) for three-dimensional digital models of *Nanhsiungchelys yangi* and two hypothetical turtles at different mesh sizes. Blue circles represent results for the *Nanhsiungchelys yangi* model, orange squares represent results for the hypothetical turtle model Ⅰ, and gray triangles represent the hypothetical turtle model Ⅱ.

Result

The CFD simulations allow us to evaluate the drag produced by each of the turtle models. In simulations with flow velocities ranging from 0.6 m s^-1^ to 2.6 m s^-1^, both the drag forces and the drag coefficients of the *Nanhsiungchelys yangi* model were always lower than the hypothetical turtle model Ⅰ (Table 2, Fig. 4). Considering the only difference between these 3-D models is whether there is a pair of anterolateral processes on carapace, this result strongly suggests that these processes played an important role in reducing resistance (~25 % reduction in drag). Moreover, the drag forces and the drag coefficients of the *Nanhsiungchelys yangi* model is close to the hypothetical turtle model Ⅱ (Table 2, Fig. 4), suggesting the former’s drag reduction ability is parallel to extant freshwater turtles. The reason for this phenomenon is the processes of *Nanhsiungchelys yangi* made the anterior part of the shell more streamlined (Fig. 5A-B), analogous to the streamlined fairing on the anterior of airplanes and rockets. The reduction of drag could enhance locomotory performance by conserving the energy expended during swimming (*Fish, 2000; Gutarra et al., 2019; Song et al., 2021*). This reinforces the importance of the anterolateral processes to the movement of *Nanhsiungchelys* in water.

Table 2:

Drag forces and drag coefficients for three-dimensional digital models of *Nanhsiungchelys yangi* and two hypothetical turtles at different flow velocities.

| Flow velocity  (m s^-1^) | *Nanhsiungchelys yangi* | | Hypothetical turtle Ⅰ | | Hypothetical turtle Ⅱ | |
| --- | --- | --- | --- | --- | --- | --- |
|  | Drag force (N) | Drag coefficient | Drag force (N) | Drag coefficient | Drag force (N) | Drag coefficient |
| 0.6 | 7.132 | 0.330 | 9.473 | 0.439 | 6.690 | 0.310 |
| 1.0 | 19.176 | 0.320 | 25.826 | 0.430 | 17.974 | 0.300 |
| 1.4 | 37.064 | 0.315 | 50.204 | 0.427 | 34.718 | 0.295 |
| 1.8 | 60.734 | 0.312 | 82.586 | 0.425 | 56.886 | 0.293 |
| 2.2 | 90.204 | 0.311 | 122.988 | 0.424 | 84.474 | 0.291 |
| 2.6 | 125.444 | 0.309 | 171.410 | 0.423 | 117.428 | 0.290 |


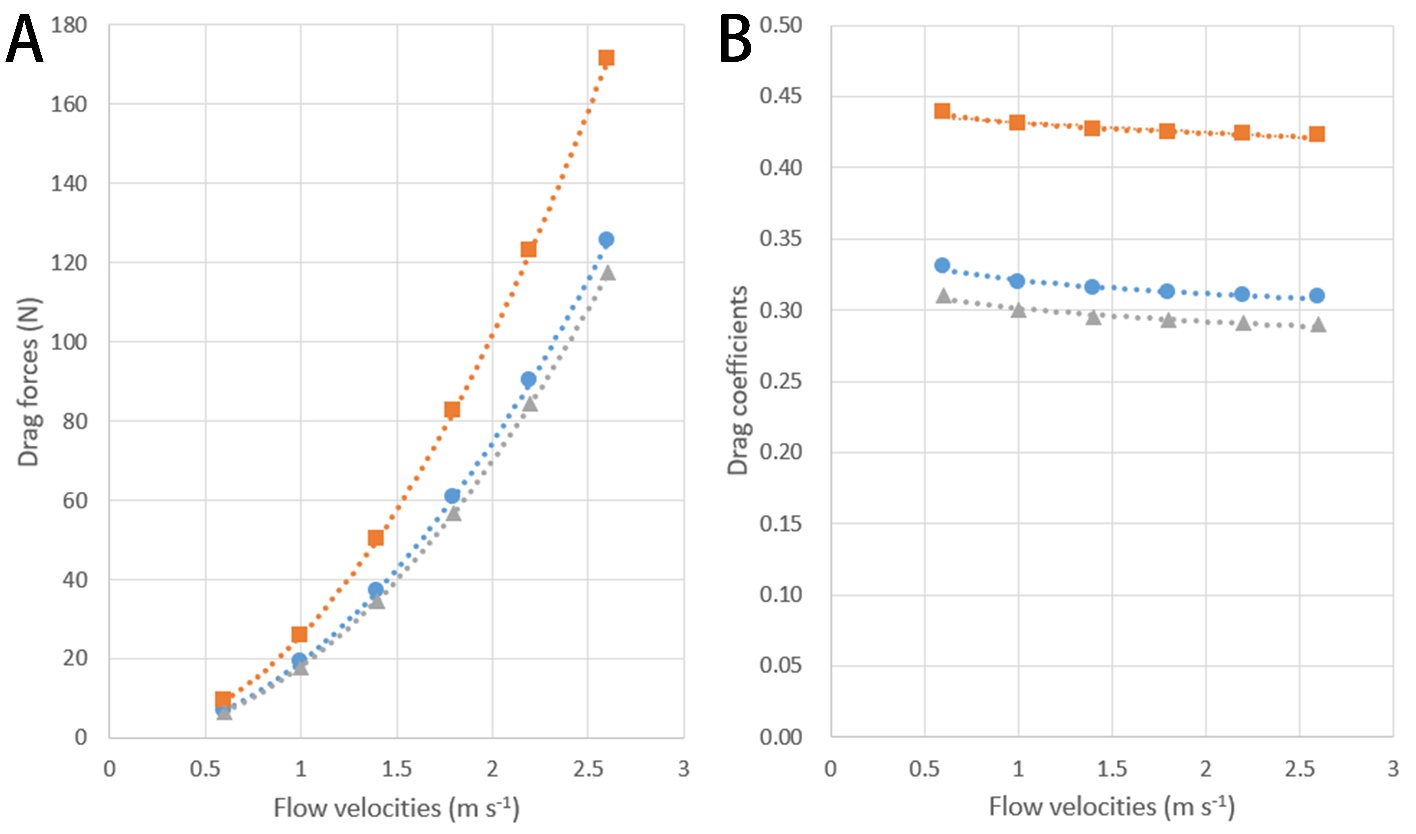


Figure 4. Comparison of drag forces (A) and drag coefficients (B) for three-dimensional digital models of *Nanhsiungchelys yangi* and two hypothetical turtles at different flow velocities. Blue circles represent results for the *Nanhsiungchelys yangi* model, orange squares represent results for the hypothetical turtle model Ⅰ, and grey triangles represent the results for the hypothetical turtle model Ⅱ.


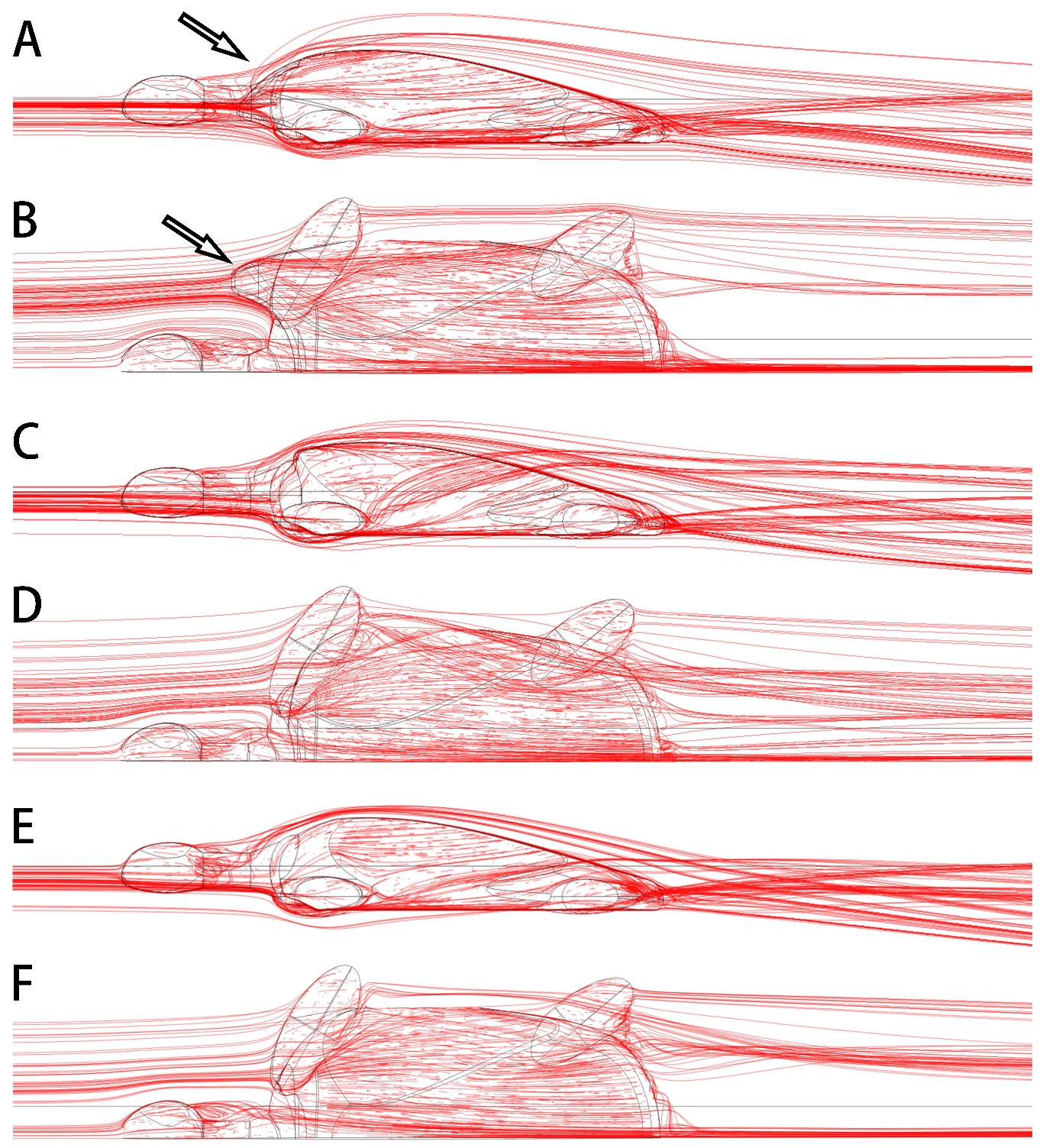


Figure 5. 3-D plots of streamlines at flow velocities of 1.0 m s^–1^. (A) and (B) are the model of *Nanhsiungchelys yangi* (in left lateral and dorsal views, respectively); (C) and (D) are the model of hypothetical turtle Ⅰ (in left lateral and dorsal views, respectively), whose anterior carapace and body are blunt; (E) and (F) are the model of hypothetical turtle Ⅱ (in left lateral and dorsal views, respectively), whose anterior carapace is streamlined and similar to most freshwater turtles. The arrows indicate the anterolateral processes. The direction of ambient flow is from left to right.

References

**Adkins D, Yan Y. 2006.** CFD simulation of fish-like body moving in viscous liquid. *Journal of Bionic Engineering* **3:**147-153. DOI:10.1016/s1672-6529(06)60018-8

**Behera S, Panda AK, Dutta SK, Nayak S. 2019.** Status survey of *Batagur baska* and *Pelochelys cantorii* in the state of Odisha, east coast of India. *Testudo* **9:**36-46.

**Dynowski JF, Nebelsick JH, Klein A, Roth-Nebelsick A. 2016.** Computational fluid dynamics analysis of the fossil crinoid *Encrinus liliiformis* (Echinodermata: Crinoidea). *PLoS One* **11:**e0156408. DOI:10.1371/journal.pone.0156408

**Eckert SA. 2002.** Swim speed and movement patterns of gravid leatherback sea turtles (*Dermochelys coriacea*) at St Croix, US Virgin Islands. *The Journal of Experimental Biology* **205:**3689–3697. DOI:10.1242/jeb.205.23.3689

**Fish FE. 2000.** Biomechanics and energetics in aquatic and semiaquatic mammals: platypus to whale. *Physiological and Biochemical Zoology* **73:**683-698. DOI:10.1086/318108

**Gibson BM, Furbish DJ, Rahman IA, Schmeeckle MW, Laflamme M, Darroch SAF. 2021.** Ancient life and moving fluids. *Biological Reviews* **96:**129-152. DOI:10.1111/brv.12649

**Guo Y, Wang J, Zhang F, Lei L, Zhao Y, Wang Y, Wang X. 2019.** A review of computational fluid dynamics and its application in quantitative analysis of the morphological function of palaeontology. *Journal of Biology* **36:**92-95. DOI:10.3969/j.issn.2095-1736.2019.05.092 (in Chinese with English abstract)

**Gutarra S, Moon BC, Rahman IA, Palmer C, Lautenschlager S, Brimacombe AJ, Benton MJ. 2019.** Effects of body plan evolution on the hydrodynamic drag and energy requirements of swimming in ichthyosaurs. *Proceedings of the Royal Society B: Biological Sciences* **286:**20182786. DOI:10.1098/rspb.2018.2786

**Gutarra S, Rahman IA. 2022.** The locomotion of extinct secondarily aquatic tetrapods. *Biological Reviews* **97:**67-98. DOI:10.1111/brv.12790

**Gutarra S, Stubbs TL, Moon BC, Palmer C, Benton MJ. 2022.** Large size in aquatic tetrapods compensates for high drag caused by extreme body proportions. *Communications Biology* **5:**1-12.

**Hirayama R, Zhong Y, Di Y, Yonezawa T, Hasegawa M. 2009.** A new nanhsiungchelyid turtle from Late Cretaceous of Guangdong, China. In: Brinkman D, ed. *Gaffney Turtle Symposium*: Drumheller: Royal Tyrell Museum, 72-73.

**Kogan I, Pacholak S, Licht M, Schneider JW, Brücker C, Brandt S. 2015.** The invisible fish: hydrodynamic constraints for predator-prey interaction in fossil fish *Saurichthys* compared to recent actinopterygians. *Biology Open* **4:**1715-1726. DOI:10.1242/bio.014720

**Liu S, Smith AS, Gu Y, Tan J, Liu CK, Turk G. 2015.** Computer simulations imply forelimb-dominated underwater flight in plesiosaurs. *PLoS Computational Biology* **11:**e1004605. DOI:10.1371/journal.pcbi.1004605

**Rahman IA. 2017.** Computational fluid dynamics as a tool for testing functional and ecological hypotheses in fossil taxa. *Palaeontology* **60:**451-459. DOI:10.1111/pala.12295

**Rahman IA, O'Shea J, Lautenschlager S, Zamora S. 2020.** Potential evolutionary trade-off between feeding and stability in Cambrian cinctan echinoderms. *Palaeontology* **63:**689-701. DOI:10.1111/pala.12495

**Reynolds O. 1883.** An experimental investigation of the circumstances which determine whether the motion of water shall be direct or sinuous, and of the law of resistance in parallel channels. *Philosophical Transactions of the Royal Society of London* **174**:935-982.

**Schaffer R, Schaffer C. 2008.** China girl’s journey: last chance for the Yangtze giant softshell turtle? *Turtle and Tortoise Newsletter* **12:**10-13.

**Shiino Y, Kuwazuru O. 2010.** Functional adaptation of spiriferide brachiopod morphology. *Journal of Evolutionary Biology* **23:**1547-1557. DOI:10.1111/j.1420-9101.2010.02024.x

**Shiino Y, Kuwazuru O, Suzuki Y, Ono S. 2012.** Swimming capability of the remopleuridid trilobite *Hypodicranotus striatus*: hydrodynamic functions of the exoskeleton and the long, forked hypostome. *Journal of Theoretical Biology* **300:**29-38. DOI:10.1016/j.jtbi.2012.01.012

**Shiino Y, Kuwazuru O, Yoshikawa N. 2009.** Computational fluid dynamics simulations on a Devonian spiriferid *Paraspirifer bownockeri* (Brachiopoda): generating mechanism of passive feeding flows. *Journal of Theoretical Biology* **259:**132-141. DOI:10.1016/j.jtbi.2009.02.018

**Song H, Song H, Rahman IA, Chu D. 2021.** Computational fluid dynamics confirms drag reduction associated with trilobite queuing behaviour. *Palaeontology* **64:**597-608. DOI:10.1111/pala.12562

**Tong H, Li L. 2019.** A revision of the holotype of *Nanhsiungchelys wuchingensis*, Ye, 1966 (Testudines: Cryptodira: Trionychoidae: Nanhsiungchelyidae). *Cretaceous Research* **95:**151-163. DOI:10.1016/j.cretres.2018.11.003

**Zhou T, Li P. 2013.** *Primary color illustrated handbook of classification of Chinese turtles.* Beijing, China: China Agriculture Press. (in Chinese)

**Zhou T, Zhou F. 2020.** *Tortoises of the World*. Beijing: China Agriculture Press. (in Chinese)
